# HIV-related non-Hodgkin lymphomas affecting the oral cavity: a clinicopathologic study of 11 cases

**DOI:** 10.4317/medoral.24993

**Published:** 2021-12-07

**Authors:** Daniel Cavalléro Colares Uchôa, Flávia Sirotheau Corrêa Pontes, Lucas Lacerda de Souza, Gabriela Sepêda dos Santos, Ana Carolina Prado-Ribeiro, Thaís Bianca Brandão, Leticia Rodrigues de Oliveira, Carolina Cavalieri Gomes, Alan Roger Santos-Silva, Felipe Paiva Fonseca, Oslei Paes de Almeida, Hélder Antônio Rebelo Pontes

**Affiliations:** 1 Oral Diagnosis Department (Pathology and Semiology), Piracicaba Dental School, University of Campinas, Piracicaba, Brazil; 2 Oral Pathology Department, João de Barros Barreto University Hospital, Federal University of Pará, Belém, Brazil; 3 Dental Oncology Service, Instituto do Câncer do Estado de São Paulo (ICESP-FMUSP), São Paulo, Brazil; 4 Department of Pathology, Biological Sciences Institute, Universidade Federal de Minas Gerais, Belo Horizonte, Brazil; 5 Department of Oral Surgery and Pathology, School of Dentistry, Universidade Federal de Minas Gerais, Belo Horizonte, Brazil

## Abstract

**Background:**

HIV-related non-Hodgkin lymphomas of the oral cavity are rare lesions with aggressive clinical behaviour. The aim of this study is to describe the clinicopathological features of a series of HIV-related oral non-Hodgkin lymphomas.

**Material and Methods:**

Eleven cases of oral lymphomas affecting HIV-positive patients were retrieved from 2012 to 2019. Clinicopathological features regarding age, sex, tumour location, clinical presentation, laboratory findings, disease stage and follow-up were obtained. Histologic, immunohistochemical and in situ hybridization for EBV detection were done for diagnosis confirmation. Overall survival was estimated by Kaplan–Meier curve.

**Results:**

Males predominated, with a mean age of 40.3 years-old. Maxilla and mandible were the mostly affected. Plasmablastic lymphoma and diffuse large B-cell lymphoma not otherwise specified (NOS) were the main histological types. Lesions presented as reddish ulcerated swellings, representing the first sign of AIDS in six cases. Stage IV were common (7 cases) and the mean HIV viral load was 10,557 copies/mL, with a mean of 266 CD4+ cells/mm3, 1,278 CD8+ cells/mm3 and a CD4+/CD8+ ratio of 0.26. Eight patients died of the disease (72.7%). Overall survival revealed that 78.2% of the patients died after 21 months of follow-up.

**Conclusions:**

HIV-related oral lymphomas present a poor prognosis usually diagnosed in advanced stages and in our series plasmablastic lymphoma was the most common subtype.

** Key words:**Oral cancer, non-Hodgkin lymphoma, HIV, survival, prognosis.

## Introduction

The human immunodeficiency virus (HIV) was initially identified in the 1980s in five young men affected by *Pneumocystis carinii *pneumonia and other opportunistic infections (1). HIV infection triggers an important immunosuppression condition by disrupting CD4+ T lymphocytes function, leading to a higher risk of developing opportunistic diseases (2,3), especially when CD4+ T cell count falls below than 200 cells/mm3, when the diagnosis of Acquired Immunodeficiency Syndrome (AIDS) is established (3,4).

The advent of the Highly Active Antiretroviral Therapy (HAART), led to a significant decrease in the incidence of the most severe systemic conditions associated with AIDS, improving the quality of life of these patients living with HIV/AIDS (3). However, some human cancers are still frequently diagnosed in HIV-positive patients (5), and they are categorized into 2 different types: 1) non-AIDS-defining cancers, including Hodgkin lymphomas, which are associated with advanced age, smoking, race and low CD4+ count (6); and 2) AIDS-defining cancers, usually associated with oncogenic viruses (7). Regarding AIDS-defining cancers, previous studies indicate Kaposi’s sarcoma as the most prevalent neoplasm, followed by non-Hodgkin lymphomas (8).

Among AIDS-associated NHL, diffuse large B cell lymphoma, primary effusion lymphoma and plasmablastic lymphoma are common subtypes, some of them frequently affecting the oral cavity, whose diagnoses may sometimes represent the clinical manifestation that leads to AIDS/HIV initial diagnosis. Hence, clinicians and pathologists must be aware of the most frequent lymphomas subtypes in the scenario of HIV infection in order to better diagnose and treat these patients. Therefore, the aim of this study is to describe the clinicopathological characteristics and the survival rate of a series of oral lymphomas affecting HIV-positive patients.

## Material and Methods

This study was approved by the Ethical Committee of the University Hospital João de Barros Barreto, Belém, Brazil (process no. 4.553.556).

All cases diagnosed as oral non-Hodgkin lymphoma between January 2012 and December 2019 were retrospectively retrieved from the pathology files of two Brazilian institutions [João de Barros Barreto University Hospital (Belém), and the Cancer Institute of São Paulo (São Paulo)]. Formalin-fixed, paraffin-embedded tissues were obtained and new histological sections were stained with haematoxylin and eosin (H&E) to be used for diagnosis confirmation by at least two oral pathologists following the current WHO classification of lymphoid neoplasms (9). The cases were analysed and those which were HIV-related were selected to be included in the final sample. The clinicopathological features were retrieved from the patients’ medical files and included age, sex, tumour location, clinical presentation, laboratory findings (CD4+, CD8+, HIV viral load and CD4+/CD8+ ratio), disease stage, treatment, status at last follow-up (dead or alive), and time of follow-up. Overall survival rate was defined as the period from the date of diagnosis to the date of the patient’s death or last follow-up. The value ranges of laboratory findings were classified following Taiwo and Hassan (10).

Immunohistochemical reactions followed the WHO recommendations to achieve the diagnosis of each case and the most appropriate primary antibody panel was specifically established for each lymphoma subtype. Briefly, reactions were performed in 3µm sections of formalin-fixed, paraffin-embedded tissues that were dewaxed with xylene and then hydrated in a descending ethanol series. Endogenous peroxidase activity was blocked with 10% hydrogen peroxide and antigen retrieval was done using citrate buffer or EDTA solution using a pressure cooker for 3 minutes. After washing in PBS buffer (pH 7.4), the sections were incubated overnight with primary antibodies, and then exposed to high-sensitivity horseradish peroxidase reagents (ADVANCE; Dako, Carpinteria, CA, USA) and diaminobenzidine tetrahydrochloride (DAB; Sigma-Aldrich, St Louis, MO, USA). The slides were counterstained with Carazzi haematoxylin for 3 minutes. Positive control histological sections were used for each antibody, while the negative control was acquired by omitting the specific primary antibody. Reactions were descriptively evaluated by at least two oral pathologists.

In order to investigate the presence of EBV, all cases were submitted to in situ hybridization (ISH) to detect the virus. A fluorescein-labelled peptide nucleic acid probe (PNA) complementary to 2 nuclear-encoded RNAs (EBER) (Y5200, Dako, Glostrup, Denmark) was hybridized at 55°C for 90 minutes, and then labelling was performed using a PNA ISH detection kit (K5201, Dako). One case of nasal-type extranodal NK/T-cell lymphoma was used as positive control. Carazzi haematoxylin was used for subsequent counterstaining. Cases considered positive for EBV presented a dark blue staining in the nuclei of the tumour cells.

The means and percentages are presented as descriptive statistics and overall survival rate was estimated by Kaplan–Meier analysis. The SPSS software version 22.0 was used for statistical analysis.

## Results

The clinicopathological features of the patients included in this study are described in Table 1. A total of 11 patients were included with a male predominance (7 cases; 63.6%) and a male: female ratio of 1.4 : 1. The mean age was 40.3 years (range 9–64 years) and the he maxilla was the most frequently affected site (6 cases; 54.5%), followed by the mandible (2 cases; 18.2%).

**Table 1:**
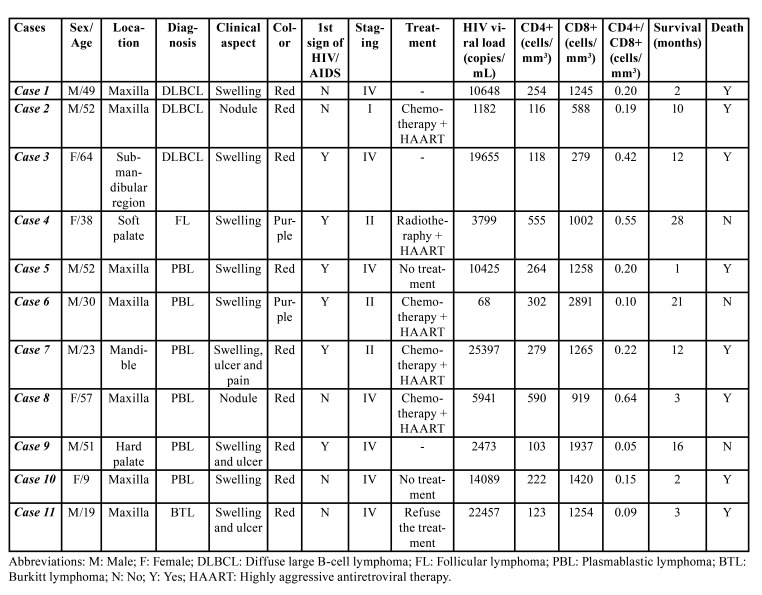
Clinicopathological features of oral HIV-related lymphomas.

**Figure 1:**
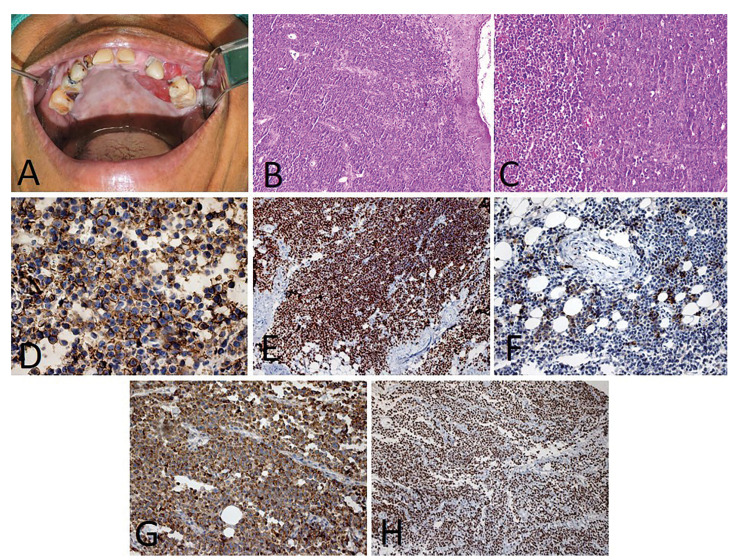
The clinicopathologic features of plasmablastic lymphoma and microscopic aspects. A) A 57-year-old female patient was presented with a bleeding swelling in the left maxilla. B) Histopathological aspects evidenced an atypical and dense infiltrate, relativity uniform, composed of large cells with a moderate cytoplasm, an eccentric nuclei, and one or more large nucleoli (H&E, 100x). C) It is also possible to see a variable number of small cells with plasmacytic appearance (H&E, 200x). The lesions showed positive immunohistochemical reactions for D) CD138 (DAB, 200x), E) MUM-1 (DAB, 200x), F) EMA (DAB, 200x), G) lambda (DAB, 200x) and H) high Ki-67 index (DAB, 200x).

**Figure 2:**
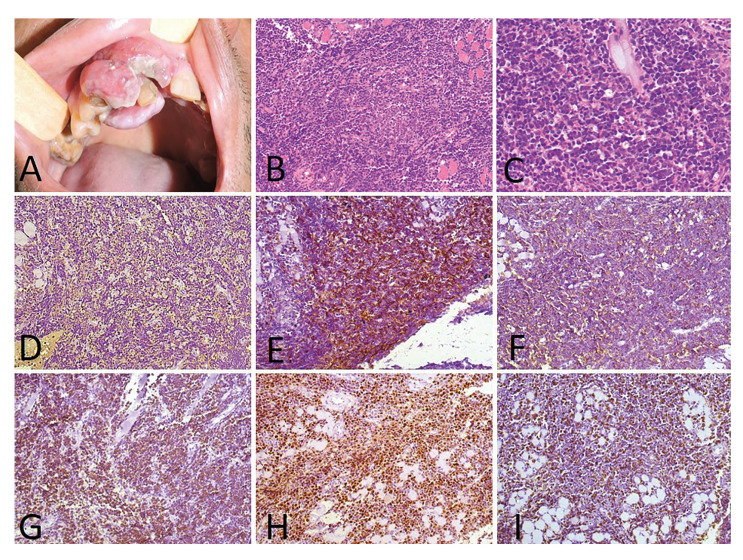
Clinicopathological features and microscopic aspects of diffuse large B-cell lymphoma. A) A 52-year-old male presented with an ulcerated gingival swelling in the anterior maxilla. B) Histopathological aspects showed sheets of predominantly medium to large mononuclear cells with pleomorphic oval to round nuclei (H&E, 100×). C) The cells were medium to large with pleomorphic oval to round nuclei (H&E, 200×). Immunohistochemical reactions evidenced positivity for D) LCA (DAB, 200×), E) CD10 (DAB, 200×), F) CD20 (DAB, 200×), G) BCL-2 (DAB, 200×), H) MUM-1 (DAB, 200×) and I) Ki-67 (DAB, 200×).

**Figure 3:**
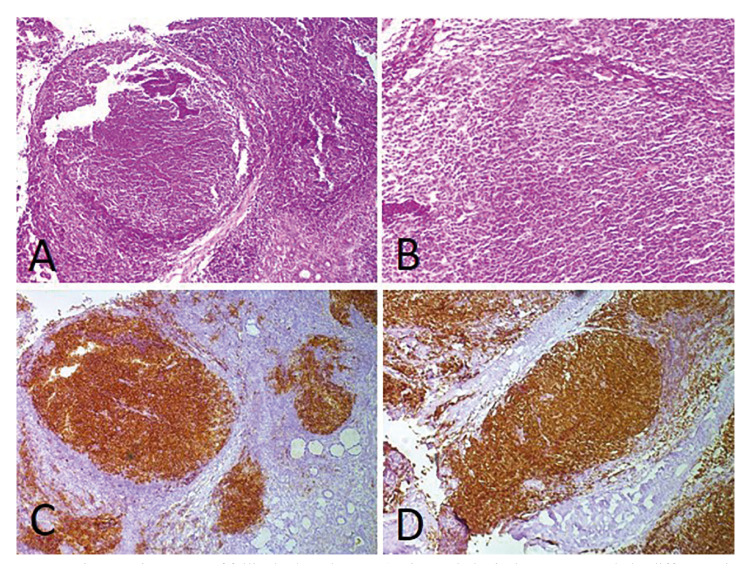
Microscopic aspects of follicular lymphoma. A) Histopathological aspects revealed a diffuse, uniform monotonous proliferation of medium-sized lymphocytes in loose fibrocellular stroma associated with follicular areas (H&E, 100×). B) Dense infiltration of centrocytes and centroblasts was observed (H&E, 200×). Positive immunohistochemical reactions for C) CD10 (DAB, 100×) and D) BCL-2 (DAB, 100×).

**Figure 4:**
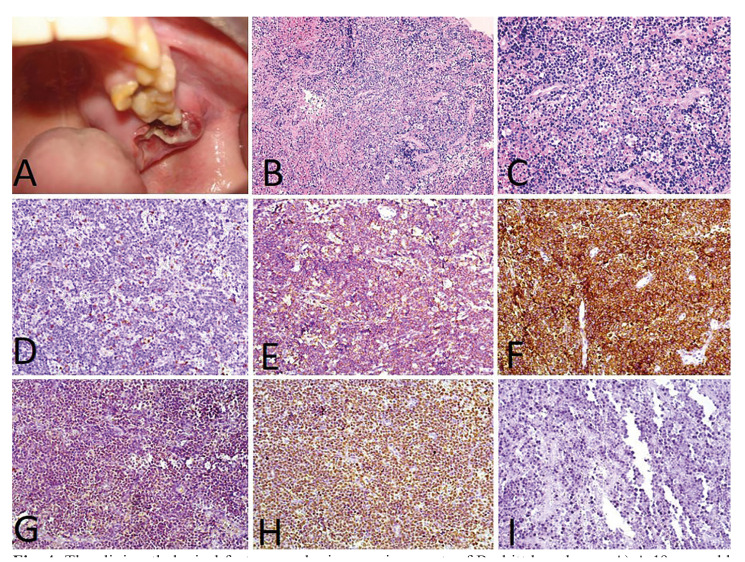
The clinicpathological features and microscopic aspects of Burkitt lymphoma. A) A 19-year-old male patients was presented with an ulcerated swelling in the posterior maxilla. B) Histopathological aspect evidenced a dense infiltrate of monotonous appearing darkly stained round cells, which appeared to be of lymphoid origin (H&E, 200x). C) On a higher magnification the tumor cells were homogenous in size and shape with round to oval intensely basophilic nuclei and minimal cytoplasm (H&E, 400x). Immunohistochemistry reactions were positive for D) CD3 (DAB, 200x), E) CD20 (DAB, 200x), F) CD10 (DAB, 200x), G) TdT (DAB, 200x), H) Ki-67 (DAB, 200x) and I) EBER (ISH, 200x).

Less commonly, the submandibular region (1 case; 9.1%), soft palate (1 case; 9.1%) and hard palate (1 case; 9.1%) were also affected. Plasmablastic lymphoma (PBL) (6 cases; 54.5%) (Fig. 1) was the most common histological type, followed by diffuse large B-cell lymphoma (DLBCL) (3 cases; 27.2%) (Fig. 2), follicular lymphoma (1 case; 9.1%) (Fig. 3) and Burkitt lymphoma (1 case; 9.1%) (Fig. 4). Clinically, the lesions predominantly presented as reddish to purple swellings (9 cases; 81.8%) or nodules (2 cases; 18.2%), with the presence of ulcers (3 cases; 27.2%). Pain was described by 1 patient only (9.1%). Oral non-Hodgkin lymphoma was the first sign of AIDS in 6 cases (54.5%). Patients mostly presented disease stage IV (7 cases; 63.6%), followed by stages II (3 cases; 27.2%) and I (1 case; 9.1%). Regarding treatment, 4 patients (36.3%) had started chemotherapy and used HAART, 2 patients (18.18%) died before starting treatment, 1 patient (9.09%) was treated with radiotherapy and HAART, and 1 patient (9.09%) refused treatment.

Laboratory findings at diagnosis showed a mean HIV viral load of 10,557 copies/mL (range 68–25,397), 266 CD4+ cells/mm3 (range 103–590 cells/mm3), 1,278 CD8+ cells/mm3 (range 279-2,891 cells/mm3) and a CD4+/CD8+ ratio of 0.26 (range 0.05–0.64).

The mean follow-up time was 10.8 months (range 1–28 months). It was observed that 8 patients died due to the disease (72.7%) and 3 were alive with no signs of recurrence (27.3%). The general overall survival revealed that 33.3% of patients were alive after 12 months, which was maintained after 21 months of follow-up. DLBCL showed a poorer survival (mean follow-up time of 8 months [range 2–12 months]) when compared with PBL (mean follow-up of 9.1 months [range 1–21 months]). The overall survival for DLBCL revealed that 0% of the patients were alive after 12 of follow-up; for PBL, 33.3% of patients were alive after 12 months of follow-up which was maintained after 21 months of follow-up.

## Discussion

Patients living with HIV/AIDS have a more than 100-fold higher risk of developing lymphomas (11), which usually manifest as asymptomatic tumors that may have necrotic regions more frequently in the palate, gingiva and alveolar mucosa (11,12), as demonstrated in our series. Moreover, B-cell origin NHL seems to predominate among patients living with HIV/AIDS and in agreement with previous reports (11,13), we also found PBL to be most common subtype in our sample, although DLBCL predominated in other series (14,15).

It is known that CD4 cell count and HIV RNA levels are directly associated with the risk of developing oral NHL (16,17,18). Our results support these findings, since the average CD4 cell count in our case series was low (266 cells/mm3), and HIV RNA was high (10,557 copies/mL). Although, it is unclear how HIV induces chronic B-cell activation with consequent somatic hypermutation, the reduction of immune surveillance allows a permissive effect for EBV to infect B cells, generating chronic B-cell stimulation, which contributes to lymphomagenesis (19). Alternatively, HIV may also produce HIV-derived p17 which promotes changes to the environment (17). Lastly, several soluble cytokines which are potent growth and antiapoptotic factors like IL-6, IL-10, sCD27, sCD44 and sCD30, have been found at elevated levels in HIV-infected individuals (16,17).

EBV contributes to the development of B-cell lymphoma by enhancing genetic instability and altering the expression of proto-oncogenes (19,20,21). Hijlkema *et al*. showed that HIV-infected individuals with EBV DNA loads above 100,000 IU/mL in plasma or serum have increased risk of developing AIDS-related lymphoma. In agreement with these observations, 8 of the 11 cases in our series were associated with EBV (all PBL and BL cases). EBV inhibits p53 gene expression and activates BCL-2, leading to radiotherapy and chemotherapy resistance through suppression of apoptosis (12,20,22).

PBL is an aggressive high-grade B-cell NHL, with a plasmacytic immunophenotype and immunoblastic morphology, commonly described in the oral cavity of HIV-infected individuals, although immunocompetent patients may also be affected (11). Boy *et al*. (13) and Alli & Meer (11) demonstrated that PBL predominated among oral lymphomas in HIV-positive patients, usually affecting males, as also shown in our series, possibly due to a higher frequency of HIV male patients. Moreover, Castillo *et al*. (23) reported that HIV-positive PBL patients were younger than HIV-negative subjects and we showed that our patients were under 60 years old, as reported previously (11).

In a recent literature review, EBV was observed in 63.4% of PBL cases, being associated with a poor prognosis. In the present series, all PBL patients were EBV-positive and it is hypothesized that HIV infection is responsible for creating conditions in which EBV can infect B cells, preventing their apoptosis (22). At the same time, it is not clear if HIV status interferes PBL prognosis. Despite its aggressiveness, Castillo *et al*. (23) showed that HIV-negative status is associated with worse overall survival, which could not be demonstrated by other authors.

DLBCL has been described as the most common subtype of NHL in HIV positive patients, demonstrating a worse prognosis in the presence of EBV (14,18,24). Although the presence of this virus is uncommon and we did not find any positive case in our series, Chao *et al*. (25) observed 30.9% positive cases in their DLBCL sample affecting HIV-positive patients, demonstrating that these patients must always be investigated for the presence of EBV. DLBCL are frequently diagnosed when patients present a poor immunological state, as demonstrated in our study where patients had an average of CD4 T cell count of 162/μL, similar to Wu *et al*. (15) in their series of 104 DLBCL in patients living with HIV and AIDS. These severely immunocompromised states used to negatively impact the survival of patients, but the use of HAART raised their survival rates to similar levels of immunocompetent individuals.

Most HIV-associated BL are EBV-negative tumors (26). Rubinstein and collaborators showed in an evaluation of 54 HIV-related BL that 48% of patients showed normal CD4+ ranges and 19% evidenced undetecTable HIV viral load (20). CD4+ T cells influence in the survival of B-cell in the germinal centre, and in long-term HIV infection presenting high CD4+ cell count may lead the disruption of germinal centres, preventing BL development (20,21). In addition, activation of c-Myc and inactivation of p53 have also been reported in AIDS-related BL (12). In a Brazilian study, HIV-associated BL more commonly affects male patients in the fourth decade of life, and also show a poor prognosis with a medium survival of 12 months (27).

Among NHL affecting the oral cavity, 8-15% are FL. The lesion in currently seen with and indolent course, although transformation to DLBCL may occurs in 20-30% of cases (28). To the best of our knowledge, it is the first case of FL in a people living with HIV/AIDS. EBV is not commonly presented in FL, and when occurs, it is mostly seen in high grade tumours (29).

In conclusion, HIV-related oral lymphomas are uncommon lesions with aggressive biological behaviour. Plasmablastic lymphoma was the most common histological type and mostly affected the maxilla. Due to the association with HIV/AIDS, the present tumours lead to advanced disease stages and poor prognosis.
